# Evolutionary history of the oriental fire‐bellied toad (*Bombina orientalis*) in Northeast China

**DOI:** 10.1002/ece3.7318

**Published:** 2021-03-05

**Authors:** Liqun Yu, Shuai Zhao, Yanshuang Shi, Fanbing Meng, Chunzhu Xu

**Affiliations:** ^1^ College of Life Science Northeast Agricultural University Harbin China

**Keywords:** amphibia, bombinatoridae, climate fluctuations, genetic diversity, geological event, population genetic structure

## Abstract

The evolutionary history of a species is generally affected by the combination of geological events and climate fluctuations. By analyzing the population features, genetic structure and the effective population historical dynamics of existing species, the population evolutionary history can be reestablished. In recent years, geological evidence shows that the Yilan–Yitong fault zone located in Northeast Asia experienced strong and frequent geological changes in the late Quaternary period. Species population history has been shaped by the combination of the complex climatic conditions of the Quaternary and Pleistocene glacial interglacial cycles and palaeogeological events in Northeast Asia and it has become a research focus for evolutionary biology researchers. In this study, mitochondrial and microsatellite molecular markers were used to reveal the population features, genetic structure, and the effective population historical dynamics of the Oriental fire‐bellied toad (*Bombina orientalis*). The results showed that the strong seismic activity of the Yilan–Yitong fault zone in the late Quaternary period was the main reason for the population differentiation of Oriental fire‐bellied toad in northeast China. The Quaternary Pleistocene glacial interglacial cycles led to the significant bottleneck effect of the western population located in the Maoer mountain area. As a result, the western population has low genetic diversity. Recent gene flow between eastern and western populations and historical evidence of population expansion proved that the dispersal behavior of the western populations was the main cause of the low genetic diversity and mitochondrial and nuclear discordance. Human economic activity may be the mainly driving factor. These evidences showed that the comprehensive influence of geology, climate, human activities and other factors should be considered in the process of exploring the evolutionary history of species.

## Graphical abstract

In this work, we use mitochondrial and microsatellite molecular markers to reveal the evolutionary history of the Oriental fire‐bellied toad (*Bombina orientalis*) in northeast China. Our study showed that the strong seismic activity of the Yilan–Yitong fault zone in the late Quaternary period was the main reason for the population differentiation of Oriental fire‐bellied toad in northeast China. Further research in this direction will provide important insights into forecasting population dynamics in the face of global change, such as the identification of key population‐specific traits for maintaining the evolutionary potential or assessing differences in population vulnerability depending on spatial patterns of biodiversity.

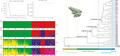

## 1 INTRODUCTION

Geological events and climate fluctuations are two important factors affecting the genetic variation pattern of species. Geological events lead to changes in the topography of the original habitat of organisms, forming obstacles such as mountains and rivers, which lead to population diffusion, reduction of gene flow between populations and result in the differentiation of a population's genetic structure (Crawford et al., 2007). Climate fluctuation also affects the genetic structure and effective size of populations. Low temperatures in the glacial epoch cause a species to retreat from high latitude to hide in the warm biological refuge of the lower latitude. Populations in different refuges lose their genetic diversity and genetic differentiation occurs because of the bottleneck effect, which is due to factors such as habitat loss, gene flow reduction, genetic drift and natural selection. During warm interglacial periods, a species's distribution will expand from the refuges to the new distribution area and change the genetic structure of the population (Hewitt, 2000, 2004). Whether these two driving factors affect the genetic variation pattern of the species population alone or together can usually be judged from the genetic imprints of the population. If geological events affect the pattern of genetic variation of species, the time of genetic lineage differentiation of species usually coincides with the time of ancient geological events. For example, the formation of the Qinghai Tibet Plateau has been proven to influence the diffusion and evolution of various biological groups (Favre et al., 2015; Klaus et al., 2016). If Pleistocene climate fluctuations affect the genetic variation of species, then the time of genetic lineage differentiation of species population usually coincides with the time of climate events and the effective population size of a species will change with the climate fluctuations (Huang et al., 2017; Yu et al., 2013). This outcome was also found in research on North America and Europe (Avise, 2000; Hewitt, 2000). The related research on the effects of the Quaternary glacial period in Northeast Asia is scarce and controversial, especially surrounding the degree and mechanism of geological events and the effects of climate fluctuations on the genetic variation of affected species (Hewitt, 2004).

**FIGURE 1:**
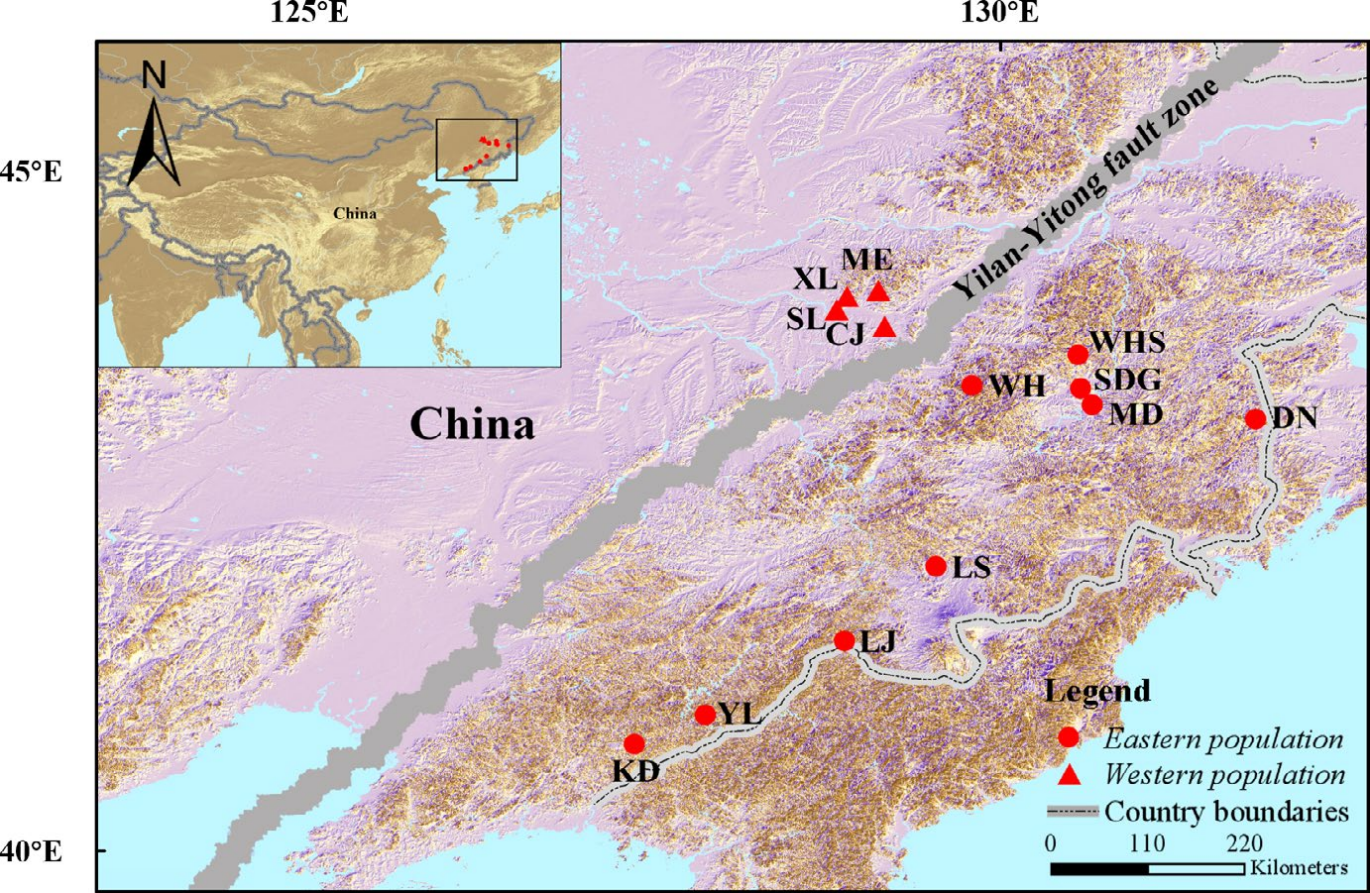
Map of all sampling sites in Northeast Asia. Detailed sampling site information is also presented in Table S1. Abbreviation for: CJ, Chenjia Village; DN, Dongning; KD, Kuandian; LJ, Linjiang; LS, Lushui River; MD, Mudanfeng; ME, Maoer Mountain; SDG, Sandaoguan; SL, Shuanglong Villa; WH, Weihe; WHS, Weihu Mountain; XL, Xiaoling; YL, Yulin Town

The climate change created by Quaternary and Pleistocene glacial interglacial cycles was an important historical factor affecting the distribution of species in Northeast Asia. Frequent climate fluctuation cause changes in the distribution of species, especially in temperature sensitive species (Dynesius & Jansson, 2000). There are two phylogenetic branches of Chinese black‐spotted frog populations (*Pelophylax nigromaculata*) in northeast China. Different branches were in refuges in different regions during the Pleistocene glacial epoch, which resulted in genetic differentiation among the lineages, but the warm and humid climate of the interglacial period led to population expansion and the two lineages had secondary contact in the eastern Liaoning Province, which resulted in strong gene flow (Zhang et al., 2008). In contrast, the study of the short‐tailed Planter (*Gloydius brevicaudus*) showed that all lineages of *G. brevicaudus* seemed to be unaffected by glacial cycles during the Late Pleistocene and vicariance patterns dominated its history (Ding et al., 2011). As a result, it is not sufficient to only consider the role of climate factors in population genetic differentiation. The Tan–Lu fault zone is the largest active seismic zone in eastern China and the Yilan–Yitong fault zone is an important branch in northeast China (Zhang et al., 2015). The Yilan–Yitong fault zone starts from Liaodong Bay in the South and extends through Shangzhi and Yilan of the Heilongjiang Province, finally reaching Luobei (Figure 1). There has been geological evidence that the Yilan–Yitong fault zone had a lot of tectonic activity in the late Quaternary period that might have affected the distribution of existing species (Yu et al., 2018). This suggests that the biological group located in the area of Yilan–Yitong fault zone may have experienced a more complex isolation and evolutionary history. We hope through the study of the Oriental fire‐bellied toad (*Bombina orientalis*) population to prove this point, and to provide a reference for other biological groups that share a sympatric distribution.

The Oriental fire‐bellied toad is an amphibian widely distributed in Northeast Asia (Fei, 2016). Fong et al. (2016) analyzed the genetic structure of the Oriental fire‐bellied toad in Northeast Asia. They found that sympatric differentiation happened in populations of northern Korea. It seems that the range overlap of the two lineages resulted from allopatry, divergence, and then secondary contact (Fong et al., 2016). Combined with the situation that northeast China was affected by quaternary glaciation and geologic events, the evolutionary history of the Oriental fire‐bellied toad was likely to be more complex. We hope to use molecular biology methods to analyze the genetic structure and evolutionary history of the Oriental fire‐bellied toad population in northeast China, and further reveal the mechanisms that have led to its existing distribution pattern.

This study will help to understand the role of geological events and climate fluctuations in affecting the genetic variation pattern of species in Northeast Asia and provide a scientific basis for proving the important role of biological refuges in the evolution of biodiversity in the glacial epoch of Northeast Asia and biological evidence for the specific active time of Yilan–Yitong fault zone.

## 2 MATERIALS AND METHODS

### 2.1 Sample collection and laboratory methods

A total of 523 specimens were sampled from 13 localities in northeast China from May to July in 2016 to 2018, based on geographical distribution and breeding season characteristics of the Oriental fire‐bellied toad (Figure 1). Sample sizes of per locality are indicated in Table S1. The samples of the western population were from Shuanglong Villa (SL), Chenjia Village (CJ), Xiaoling (XL) and Maoer Mountain (ME). These locations were distributed around Maoer mountain in the Heilongjiang province and on the western side of the Yilan–Yitong fault zone. The samples of the eastern population were from Weihen (WH), Weihu Mountain (WHS), Sandaoguan (SDG), Mudanfeng (MD), Dongning (DN), Lushui River (LS), Linjiang (LJ), Yulin Town (YL) and Kuandian (KD). These sites were located on the eastern side of the Yilan–Yitong fault zone.

Toe tissue clips were collected for all individuals. All individuals were released back into the wild post tissue collection. The wounds were disinfected with a pain relieving, antiseptic and antibacterial Bactine® spray before being released at the capture site. Samples were stored in 95% ethanol. This study protocol was approved by the Ethics and Experimental Animal Committee of Northeast Agricultural University, China.

The DNA from toe tissue samples was extracted using the phenol‐chloroform extraction method (Sambrook & Russell, 2001). The protocol of Shi et al. (2018) was followed for microsatellite polymorphism primer information and PCR amplification process to obtain relevant data of 12 microsatellite loci in all individuals. The microsatellite loci were verified by three methods: (a) using Genepop v.4.0.10 (Raymond & Rousset, 1995) software to estimate null allele frequencies, (b) *p*‐Value of Hardy–Weinberg equilibrium (*H*_we_), and (3) linkage disequilibrium (LD) of 12 microsatellite loci. The *H*_we_ and null allele frequencies tests were performed for each microsatellite loci in each sample site. Tests for LD were performed based on each pair of microsatellite loci. A sequential Bonferroni method was used to calibrate the *p*‐values. The microsatellite loci with no high null allele frequency conformed to *H*_we_ and without linkage inheritance in each population were selected to further determine the genetic structure and diversity of populations.

The specific primers used to amplify mitochondrial cytochrome c oxidase subunit I (*COI*) and mitochondrial cytochrome b (*Cytb*) genes were based on the *COI* and *Cytb* sequences published on GenBank and designed using Primer Premier v.5.0 (Singh et al., 1998). Here *COI* was amplified using forward primer (5’‐CAAATCACAAAGACATTGGCACCCT‐3’) and reverse primer (5’‐GATACGACATAGTGGAAGTGGGCTAC‐3’). The *Cytb* was amplified using forward primer (5’‐GTATGTCACCCAACCTCCGAAAATC‐3’) and reverse primer (5’‐CAACTGGTTGTCCTCCAATTCATG‐3’).

At least ten samples were selected from each sampling point for mitochondrial gene amplification. These PCRs were conducted in 25 µl reaction systems. The PCR cocktail (TaKaRa, Beijing, China) included 20ng of template DNA, 0.2 µl Taq DNA polymerase (5 U/µl), 1 µl MgCl2 (25 mM), 1µl dNTP Mixture (2.5 mM each), and 0.80 µl of each primer (10 µM) in 2.5 µl 10 × PCR buffer. All PCRs were conducted with the following reaction profile of 5 min for initial denaturation at 95°C, followed by 30 cycles of 95°C denaturing for 1 min, 56°C annealing for 1 min, 72°C extension for 1 min and a final additional extension step at 72°C for 10 min. PCR products were sequenced in both directions using an ABI3730XL machine (Applied Biosystems). Complete sequences were compared visually to the original chromatograms to avoid reading errors. Sequences were aligned manually with BioEdit v.7.0.9 (Hall, 1999).

### 2.2 Population genetic structure analysis

Nuclear genes and mitochondrial genes were used to analyze the genetic structure of Oriental fire‐bellied toad population in northeast China. Genealogical relationships among mitochondrial DNA (mtDNA) haplotypes were constructed using median‐joining networks with Network v.10.1 (Bandelt et al., 1999). To assess genetic clustering of Oriental fire‐bellied toad population, we used a Bayesian inference with the program Structure v.2.3.3 (Pritchard et al., 2000), assuming an admixture model with correlated allele frequencies model (λ = 1). *K* values between 1 and 10 were examined. Each *K* value was set to run ten independent simulations and a burn‐in period of 10^6^ iterations followed by 10^5^ Markov Chain Monte Carlo (MCMC) iterations. The posterior probability of *K* from each independent run was used to infer the optimum number of clusters. As the ∆*K* method of Evanno et al. (2005) does not allow *K* = 1 to be tested, this method was employed when *K* was higher than one for the log‐likelihood using Structure Harvester (Earl & VonHoldt, 2012).

### 2.3 Estimation of divergence dating

The software BEAST v.1.8.2 was used to estimate divergence dates based on mtDNA haplotypes (Drummond & Rambaut, 2007). A strict clock model was implemented with MCMC chains run for 10^7^ generations and sampled every 1,000 generations. A GTR (general time reversible) + I + G substitution model was used with a Yule process tree prior. We set priors for calibration points based on the divergence between *B.bombina* (GenBank accession number: JX893173, JX893173) and *B.variegata* (GenBank accession number: AY971143, AY971143) following a previous study of *Bombina* (Nurnberger et al., 2016). We implemented a normal distribution with a mean of 3.6 million years ago (Mya), *SD* of 0.24 and 95% highest posterior density (HPD) of 3.3–3.9 Mya. Tracer v.1.6 (Drummond et al., 2012) was used to investigate performance with a 10% burn‐in and tree data were summarized in TreeAnnotator to generate a consensus tree.

### 2.4 Population genetic diversity analyses

Combined with the results of population genetic structure analysis, nuclear genes and mitochondrial genes were used to analyze the population genetic diversity of the detailed population. Based on mitochondrial genes, polymorphic sites (*S*), number of haplotypes (*H*), haplotype diversity (*H*_d_), nucleotide diversity (*π*) and average number of nucleotide differences (*K*) were estimated with DnaSP v.5.0 (Librado & Rozas, 2009). Using the verified microsatellite loci, genetic diversity was estimated for each sample site from mean number of alleles (*N*_a_), mean number of effective alleles (*N*_e_), expected heterozygosities (*H*_e_) and observed heterozygosities (*H*_o_) in GenAlEx v.6.5 software (Peakall and Smouse, 2012).

### 2.5 Population historical dynamics

To understand the recent historical dynamics of Oriental fire‐bellied toad population in northeast China, we tested for a bottleneck effect using the microsatellite data, and neutral tests and mismatch distributions were performed on the mitochondrial data. The three models (SMM: stepwise mutation model, TPM: two phase model of mutation, IAM: infinite allele model) in Bottleneck v1.2.2 (Piry et al., 1999) were used for the analysis of Sign test, standardized differences test and Wilcoxon signed‐rank test. The TPM variance was set to 10%, the proportion of SMM to TPM to 5% and run 10^4^ times repeatedly. The three different models and detection standards were used to determine whether Oriental fire‐bellied toad population experienced the bottleneck effect. Tajima's D and Fu's Fs tests were performed in Arlequin v3.5 (Excoffier & Lischer, 2010) and were used to detect whether the allele variation frequency of Oriental fire‐bellied toad populations deviated from the neutral evolutionary model. The mismatch distribution test was performed in Arlequin software and the bootstrap was set to 1,000.

### 2.6 Population gene flow

Using microsatellite and mitochondrial data, respectively, we estimated the possibility of gene flow among Oriental fire‐bellied toad populations. The effective migrating number of individuals in each generation (M) between populations in different regions were estimated by the Bayesian algorithm implemented in Migrate‐n v 4.4.3 (Beerli & Palczewski, 2010). For microsatellite data, we selected a Brownian motion model, we set the number of recorded steps in chain and burn‐in for each chain to 100,000 and 10,000, respectively, and other parameters used were default. For mitochondrial data, the number of recorded steps in chain and burn‐in for each chain were set to 10,000 and 1,000, respectively, with the default set used for all other options.

## 3 RESULTS

### 3.1 Data acquisition and assessment

523 samples of microsatellite data, and 139 mtDNA *COI* (903 bp) and *Cytb* (885 bp) sequences were obtained. The results of LD test showed that there was no LD across any pair of loci. The null allele frequency of loci 19, 141 and 23 in each sampling site was significantly higher than that in other loci (Table S2). Among them, locus 19 in 8 sampling sites, locus 141 in 10 sampling sites, and locus 23 in 7 sampling sites had significant deviations from *H*_we_ (*p* < 0.01) (Table S3). Therefore, we removed these three loci in the subsequent population genetic structure and diversity analyses based on microsatellite data.

### 3.2 Population genetic structure and estimation of divergence dating

**FIGURE 2:**
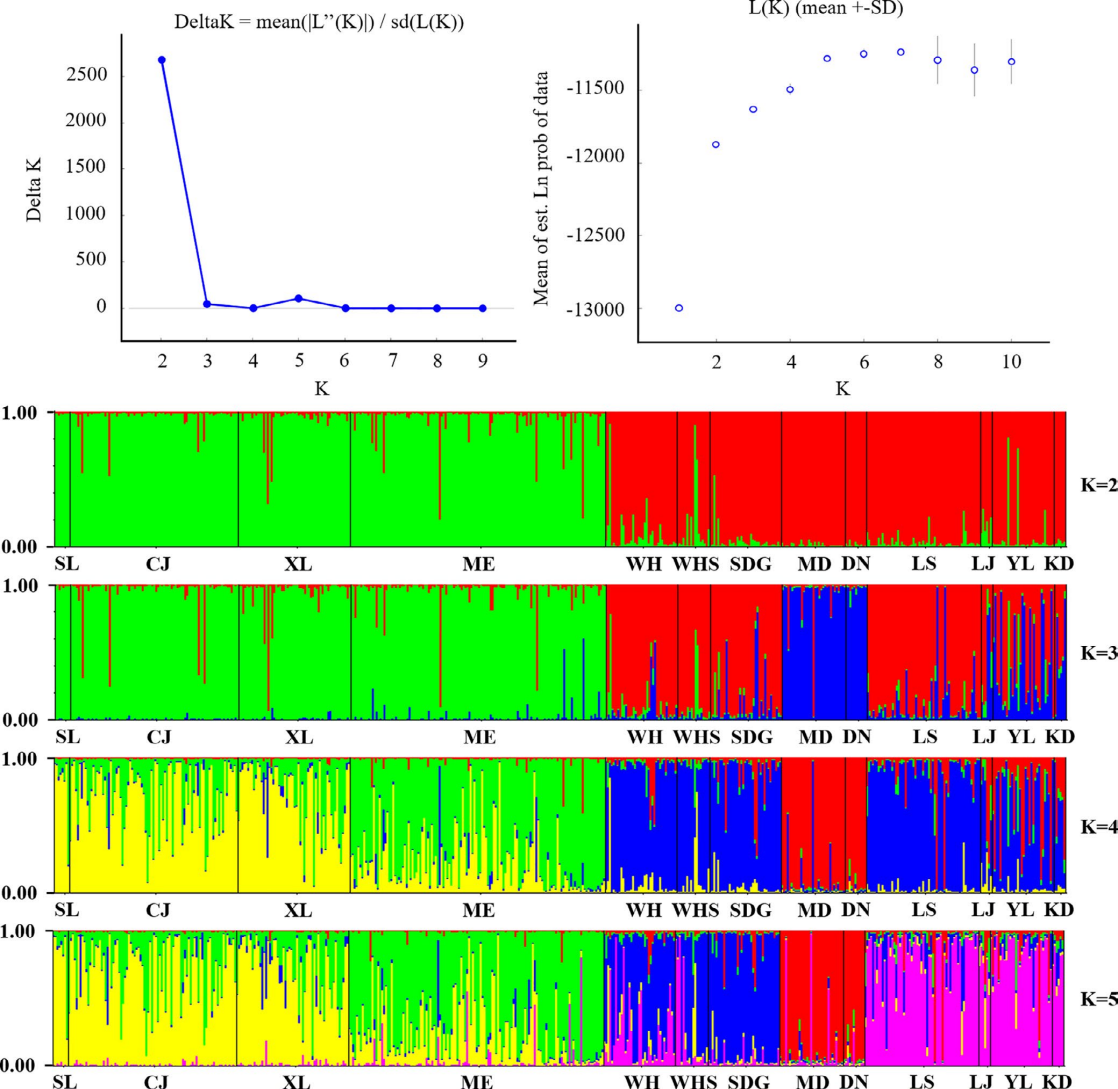
Population structure and individual assignment of Oriental fire‐bellied toad, assessed by Bayesian clustering of microsatellite DNA using Structure for *K* = 2–5

**FIGURE 3:**
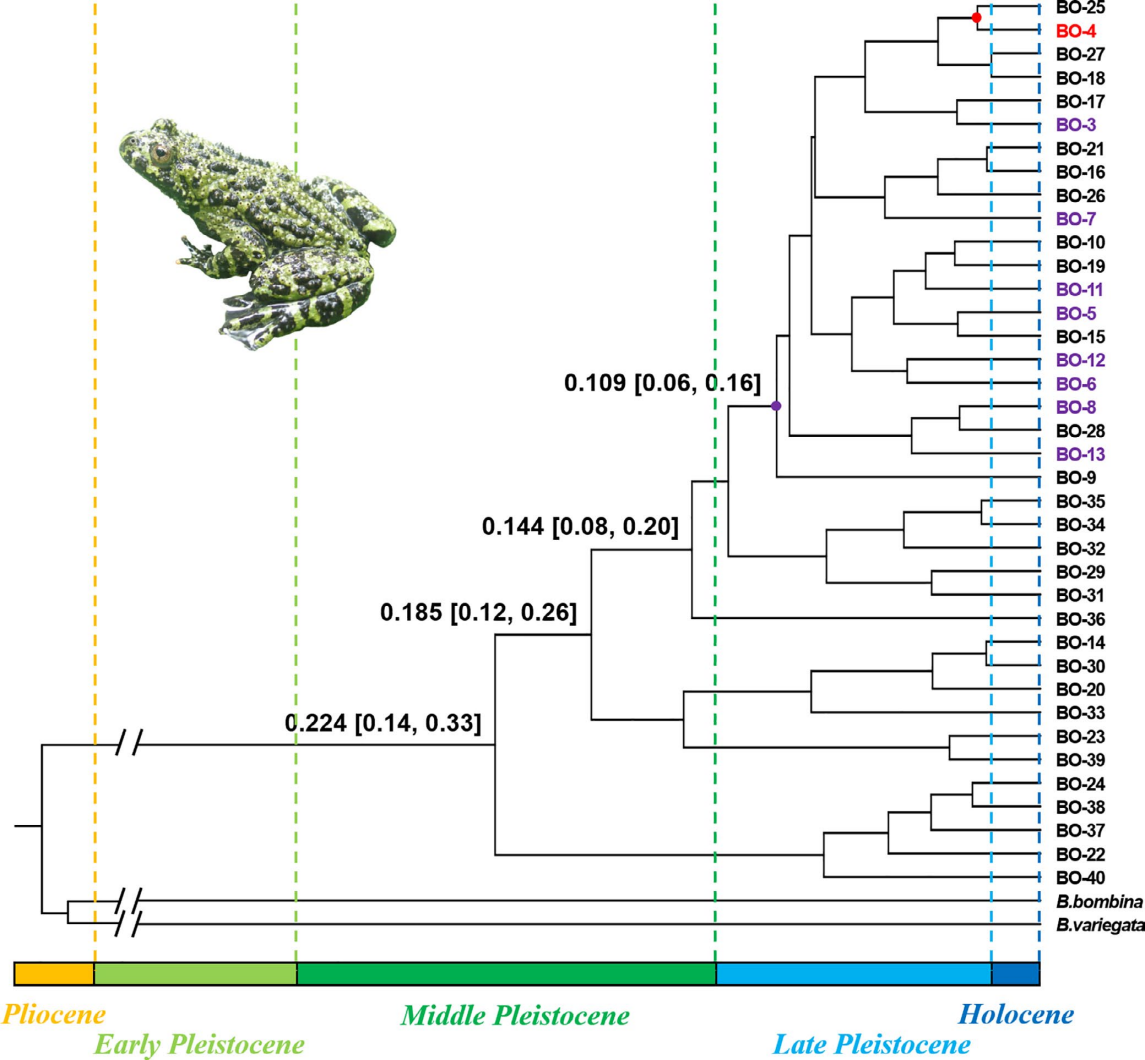
Differentiation time estimates based on mitochondrial DNA, median estimates of node ages for major differentiation events are labeled on the tree. The labels marked purple are haplotypes belong western population. The label marked red is the shared haplotype between two populations

**FIGURE 4:**
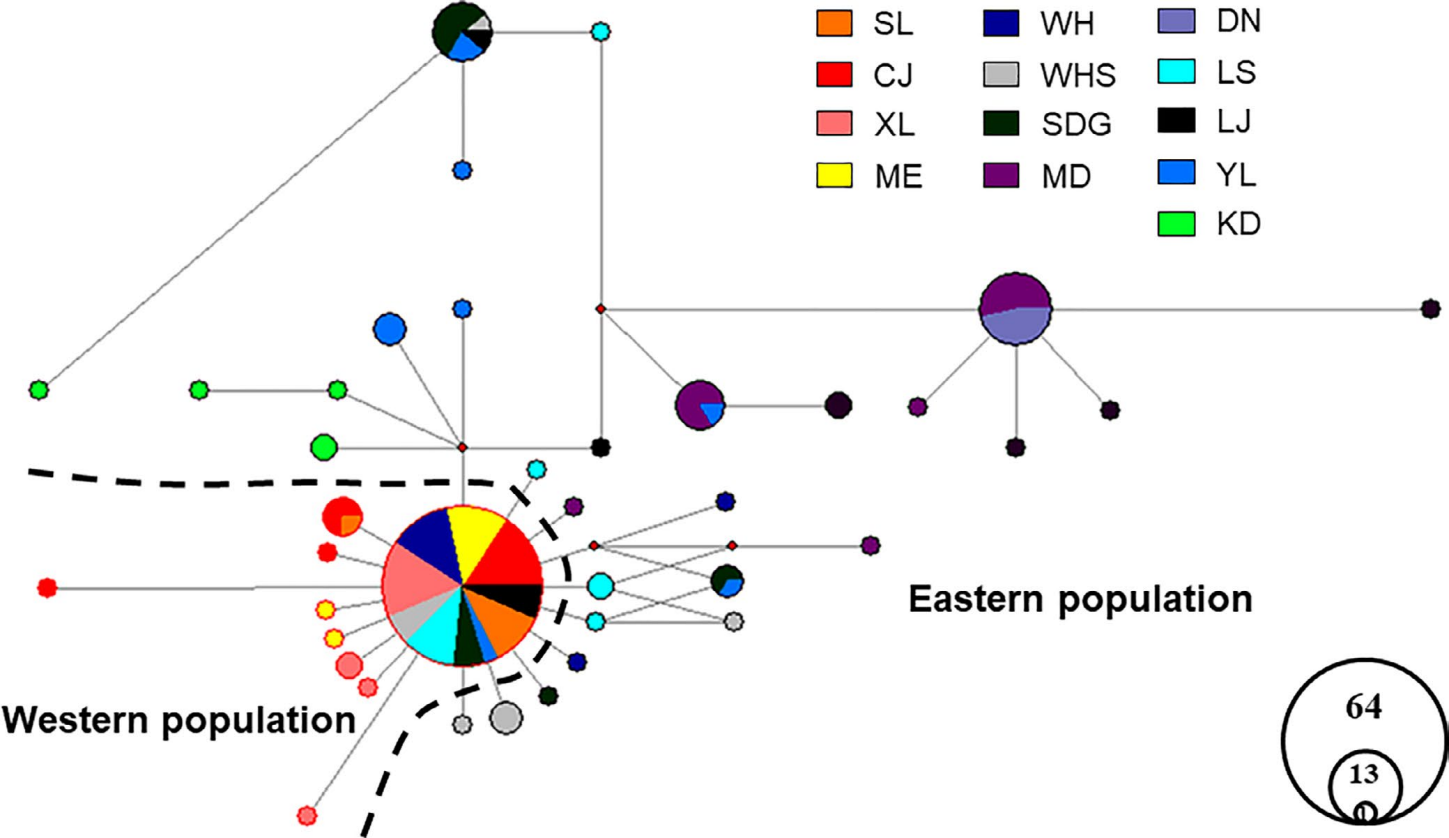
The haplotypes network of Oriental fire‐bellied toad. Different color means different sampling sites. A circular node represents a haplotype. Small dot between nodes represents lacked haplotype. The circle size represents the number of individuals included (The biggest circle includes 64 individuals)

To identify the number of naturally occurring clusters (*K*) among the 13 sample sites, the program Structure was used with microsatellite DNA. Our analysis revealed that the microsatellite DNA were divided into two populations (*K* = 2), with ∆*K* value at this time as the largest. As described earlier, cluster I incorporated the western population and cluster II incorporated the eastern population (Figure 2). Based on mitochondrial haplotypes constructed the Bayesian time tree showed that the western population diverged from the eastern population at 0.109 Mya (95%HPD 0.06–0.16) (Figure 3). Haplotype network analysis also showed that there were two populations, but there was a shared haplotype between them (Figure 4). The shared haplotype BO‐4 (0.029 Mya, 95%HPD 0.0006–0.0611) diverged more recently (Figure 3). Each major clade is strongly supported by the posterior probabilities from the Bayesian inference.

### 3.3 Population genetic diversity

The results of populations genetic diversity calculation based on microsatellite and mitochondrial gene data are shown in Table 1. All 139 mtDNA samples were used for genetic diversity analysis. For Oriental fire‐bellied toad in northeast China, the number of haplotypes (*H*) was 38, number of polymorphic sites (*S*) was 42, haplotype diversity (*H*_d_) was 0.774, nucleotide diversity (*π*) was 0.00182 and the mean number of nucleotide differences (*K*) was 3.245. The level of genetic diversity of the eastern population was significantly higher than that of the western population. In general, Oriental fire‐bellied toad in northeast China showed high haplotype diversity and low nucleotide diversity (*H*_d_: 0.774; *π*: 0.00182). The western population showed low haplotype diversity and low nucleotide diversity (*H*_d_: 0.443; *π*: 0.00035) and the eastern population was the same as the overall level, showing high haplotype diversity and low nucleotide diversity (*H*_d_: 0.869; *π*: 0.00226). The genetic diversity of each sample site is shown in Table S4.

**TABLE 1:** Statistics of genetic diversity indices based on mitochondrial and microsatellite DNA

	Western population	Eastern population	All
Mitochondrial DNA			
*H*	9	30	38
*S*	11	32	42
*H*_d_	0.443	0.869	0.774
*Π*	0.00035	0.00226	0.00182
*K*	0.625	4.038	3.245
microsatellite DNA			
*N*_a_	6.778	10.444	8.611
*N*_e_	2.593	4.827	3.710
*H*_o_	0.463	0.617	0.540
*H*_e_	0.510	0.710	0.610

Abbreviations: *H*, number of haplotypes; *H*_d_, haplotype diversity; *H*_e_, expected heterozygosity; *H*_o_, observed heterozygosity; *H*_we_, *p*‐Value of Hardy–Weinberg equilibrium test; *K*, average number of nucleotide differences; *N*_a_, mean number of alleles; *N*_e_, mean number of effective alleles; *S*, number of polymorphic sites; *π*, nucleotide diversity.

The mean number of alleles (*N*_a_) and effective alleles (*N*_e_), observed heterozygosity (*H*_o_) and the expected heterozygosity (*H*_e_) were used as indicators to measure the genetic diversity based on 9 polymorphic microsatellite loci of 523 individuals from 13 sample sites. For the overall population, *N*_a_ was 8.611, *N*_e_ was 3.710, *H*_o_ was 0.540 and *He* was 0.610. The western population (*H*_o_: 0.436, *H*_e_: 0.510) had a lower genetic diversity than the eastern population (*H*_o_: 0.617, *H*_e_: 0.710). The genetic diversity of each sample site is shown in Table S5.

### 3.4 Population historical dynamics

The bottleneck effect test results are shown in Table 2. The *p*‐value in the table indicates the significance of the population's deviation from the catastrophe drift balance under the corresponding model. The eastern population had a significant *p*‐value in the three models (IAM, TPM, SMM), indicating that it had experienced the bottleneck effect, while the western population had a significant *p*‐value in all models except the IAM model, also showing that it had experienced the bottleneck effect.

**TABLE 2:** The result of Bottleneck test. Significant results are in bold

pop	IAM			TPM			SMM		
	Sign test	Standardized differences test	Wilcoxon test	Sign test	Standardized differences test	Wilcoxon test	Sign test	Standardized differences test	Wilcoxon test
Western population	0.08722	0.05677	0.15137	0.11034	**0.02348** ^*^	0.20361	**0.02268** ^*^	**0** ^**^	**0.01050** ^*^
Eastern population	0.36554	**0.00632** ^**^	**0.01053** ^*^	0.37597	**0.00149** ^**^	0.42383	**0.00351** ^**^	**0** ^**^	**0.01343** ^*^

* <0.01

** <0.001

**FIGURE 5:**
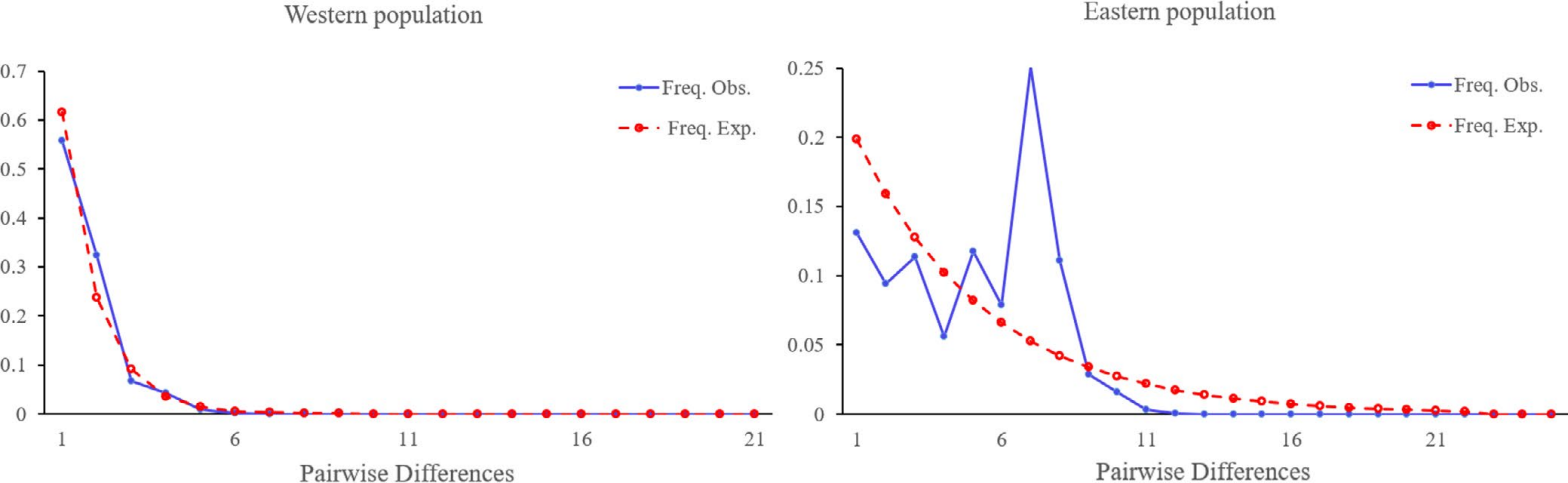
The mismatch distribution test of two populations

The western population showed a unimodal Poisson distribution in the mismatch distribution curve (Figure 5), and the values of Tajima’D and Fu's Fs were −2.205 (*p* < 0.01) and −6.831 (*p* < 0.01) respectively, indicating that there was expansion event in the recent history of the western population. The eastern population showed a multi‐peak distribution in the mismatch distribution curve, and the values of Tajima' D and Fu's Fs were −1.104 (*p* = 0.115) and −14.164 (*p* < 0.01), indicating that the eastern population was stable in the recent history.

### 3.5 Population gene flow

Migrate was used to analyze gene flow and migration between the eastern and western populations of Oriental fire‐bellied toad. The results showed that there was symmetrical gene flow of mitochondrial genes between the two populations (M_west→east_ = 390.622 ≈ M_east→west_ = 394.758), while the nuclear genes mainly flowed from the western population to east (M_west→east_ = 3.175 > M_east→west_ = 0.427). The gene flow value of the mitochondrial genes was significantly higher than that of nuclear genes.

## 4 DISCUSSION

### 4.1 Mechanism of population differentiation in oriental fire‐bellied toad

Abundant geological and climatic events at the end of the Pleistocene influenced the biogeographic patterns of many species in the present age (Miranda et al., 2018). Based on microsatellite and mitochondrial data, the genetic structure of the existing Oriental fire‐bellied toad populations in the northeast China was analyzed. This study found that there were two distinct populations with the Yilan–Yitong fault zone, which runs from northeast to southwest as a geographical barrier. The eastern population covers most of the Changbai mountain, which is on the east side of the fault zone and the western population is distributed around Maoer mountain. The intense activity of the Yilan–Yitong fault zone spanned the last glacial maximum from the last interglacial to the middle Holocene (Shi et al., 2012; Yu et al., 2018), which is consistent with the divergence time when the Western and Eastern populations split. Therefore, we believe that this geological event is an important factor affecting the genetic differentiation of these populations. In the study of Fong et al. (2016), the population of Oriental fire‐bellied toad in northeast China and in northern Korea was clustered into one lineage. The Korean Peninsula was the largest glacial refuge in Northeast Asia. Therefore, we speculate that before the arrival of the Quaternary glacial epoch, the Oriental fire‐bellied toad was widely distributed in Northeast Asia with suitable climate, while the arrival of the glacial epoch caused a sudden drop in temperature in northeast China (Liu, 1988), causing the Oriental fire‐bellied toad, which as a poikilotherm is very sensitive to the climatic conditions, to move southward immediately. But the Oriental fire‐bellied toad that is distributed west of Yilan–Yitong fault zone was affected by the strong barrier of the seismic belt, which forced it to stay in the area around the Maoer mountain. When the regional climate is no longer suitable for survival, small‐scale climate refuges provide the possibility of preserving species (Dinis et al., 2019; Worth et al., 2014). From the perspective of the existing phylogeographic pattern of the Oriental fire‐bellied toad, it is likely that there was a small‐scale climate refuge in the last glacial maximum in the Maoer mountain area. It provides the possibility for the survival of the western population, which had undergone independent differentiation after geographical isolation and genetic drift brought on by the glacial epoch, forming the currently existing pattern.

### 4.2 Changes in western population of oriental fire‐bellied toad

Intraspecific genetic diversity can not only affect the ecological dynamics of a population (Raffard et al., 2019), but also reflect the major events experienced by a species in the process of historical evolution. Based on microsatellite and mitochondrial data, this study found that the level of genetic diversity of the western population was lower than that of the eastern population. Especially in the western population, the nucleotide diversity of mtDNA was very low (π = 0.00035). This, together with the results of the bottleneck test, inferred that the western population had experienced a serious bottleneck effect. Combined with the history of the western population, even if there were small biological climate refuges, severe climate conditions in the glacial epoch may have led to the emergence of a bottleneck effect and a large amount of genetic variation would be lost in this period. Zhang et al. (2008) has reported the same situation in Chinese Black‐Spotted Frog populations. The lineage A2 of Black‐Spotted Frog populations, which is located in south‐western plateau was able to survive in lower elevations in ice‐ages and the genetic diversity of the A2 lineage was the lowest among the existing lineages. The results of our population historical dynamics analyses showed that the western population has expanded in recent years. The results of our gene flow analyses also showed that there was gene exchange between the eastern and western populations in which the gene flow of mitochondrial data was almost symmetrical, while that of microsatellite data was from west to east. There was also a shared haplotype formed recently (the formation time was later than the differentiation time of the western population) between the two populations. The Yilan–Yitong fault zone has been in‐active since the middle Holocene (Yu et al., 2018), and the isolation caused by geological events and glacial epoch has disappeared. Based on the above information, it can be inferred that the western population is dispersing into the eastern population. The main reason for this is most likely to be the habitat fragmentation caused by the increasing human economic activities (Weihmann et al., 2019).

### 4.3 Mitochondrial and nuclear discordance of the oriental fire‐bellied toad

Based on the microsatellite data, the western population can be clearly divided into a single cluster, while the haplotype network and the Bayes tree based on mitochondrial genes showed that the western population branches were mixed with samples from the eastern population and there were no samples from the western population in other branches. When analyzing the phylogeny of species respectively based on mitochondrial and nuclear genetic markers, the phenomenon of conflicting population geographical patterns is called mito‐nuclear discordance, which has been found and reported in many species (Toews & Brelsford, 2012). There were three possible reasons for the mito‐nuclear discordance of Oriental fire‐bellied toad population. Firstly, the mitochondrial genome is haploid and uniparentally inherited in most animals, and therefore has a fourfold smaller effective population size. mtDNA will complete the process of lineage sorting, where ancestral polymorphisms are lost over time, faster than nuDNA. The inheritance properties of mtDNA make it more likely than any single nuclear marker to accurately reflect recent divergence (Zink & Barrowclough, 2010). The mito‐nuclear discordance of Oriental fire‐bellied toad population can be explained by the re‐gene exchange among populations after isolation. Secondly, there was sex‐biased dispersal in Oriental fire‐bellied toad population, and the sex‐biased migration would lead to the biased movement and enrichment of specific types of genetic markers. Currat et al. (2008) predicted that gene flow of mtDNA in systems with male‐biased dispersal will be higher as compared to those systems with female‐biased dispersal (Petit & Excoffier, 2009). We also found excessive mitochondrial gene flow in Oriental fire‐bellied toad, which suggested that it may have male‐biased dispersal. In addition, the effect of mating system on gene flow has also been concerned by many scholars (Pickup et al., 2019). In the future, we may conduct more in‐depth studies on the mating system and dispersal patterns of Oriental fire‐bellied toad population. Thirdly, incomplete lineage sorting (ILS) is one of the most well‐described causes of mito‐nuclear discordance (Firneno et al., 2020). It is not enough to only use Structure with microsatellite loci to prove that the ILS led mito‐nuclear discordance of Oriental fire‐bellied toad population. We need more nuclear DNA or whole genome SNPs data to reconstruct the phylogenetic relationship of the Oriental fire‐bellied toad to determine whether ILS is the cause of mito‐nuclear discordance (Firneno et al., 2020; Toews & Brelsford, 2012).

## 5 CONCLUSIONS

The strong activity of the Yilan–Yitong fault zone from the last interglacial to the Middle Holocene was most likely the main reason for the differentiation of the Oriental fire‐bellied toad into two populations in northeast China. The Maoer mountain area was inferred to be a small‐scale climate refuge for the western population in the Last glacial maximum, which maintained it after the bottleneck effect of glacial epoch. Until recently, the western population dispersed to the east due to human activities in Maoer mountain area. In addition, we also found evidence for the mitochondrial and nuclear discordance in Oriental fire‐bellied toad populations.

## CONFLICT OF INTERESTS

The manuscript has not been published before and is not being considered for publication elsewhere. All authors have contributed to the creation of this manuscript for important intellectual content and read and approved the final manuscript. We declare there is no conflict of interest.

## AUTHOR CONTRIBUTIONS

**Liqun Yu:** Conceptualization (lead); Data curation (equal); Formal analysis (equal); Investigation (equal); Visualization (equal); Writing‐original draft (lead); Writing‐review & editing (lead). **Shuai Zhao:** Data curation (equal); Formal analysis (equal); Investigation (equal). **Yanshuang Shi:** Investigation (equal); Validation (equal). **Fanbing Meng:** Investigation (equal); Validation (equal). **Chunzhu Xu:** Conceptualization (lead); Funding acquisition (lead); Project administration (lead); Resources (lead); Supervision (lead); Writing‐review & editing (lead).

## Supporting information

Table S1‐S5

## Data Availability

All newly acquired sequences have been deposited in GenBank^®^ repository (http://www.ncbi.nlm.nih.gov) under accession numbers MK609566‐MK609843 (see Table S1). Microsatellite DNA data has uploaded as online supporting information (https://datadryad.org/stash/share/dSXduyrTUZnDZeg6hOO7oN3S4bigmmAxZbk_WF2jRh8).
