# Supplementary material for: Evolutionary history of the oriental fire‐bellied toad (Bombina orientalis) in Northeast China
Source: Ecol Evol. 2021 Mar 5;11(9):4232–42. doi: 10.1002/ece3.7318 (PMC8093726; doi:10.1002/ece3.7318)
Supplement: Supplementary file 1 — Table S1‐S5 [file ECE3-11-4232-s001.docx]

**Appendix**

**Supplementary Table S1.** Locality information, sample information, and GenBank accession number of specimens. Sample size: numbers in < > are sample sizes subjected to mtDNA sequences; the other numbers are sample sizes of microsatellite DNA.

**Supplementary Table S2.** The null allele frequencies for each microsatellite loci in each sample site.

**Supplementary Table S3**. The P-value of Hardy-Weinberg equilibrium for each microsatellite loci in each sample site.

**Supplementary Table S4**. Statistics of genetic diversity indices based on mitochondrial DNA. *H*, number of haplotypes; *S*, number of polymorphic sites; *Hd*, haplotype diversity; *π*, nucleotide diversity; *K*: average number of nucleotide differences.

**Supplementary Table S5**. Statistics of genetic diversity indices based on microsatellite DNA. *Na*, mean number of alleles; *Ne*, mean number of effective alleles; *Ho*, observed heterozygosity; *He*, the expected heterozygosity; *Hwe*, P-value of Hardy-Weinberg equilibrium test.

Supplementary Table S1

| Location name | Abbreviation | Coordinates | Sample number | GenBank accession number | Sample sizes |
| --- | --- | --- | --- | --- | --- |
| Shuanglong Villa, Heilongjiang, China | SL | 127.08°E 45.07°N | BOC133~140 | MK609569, MK609623~MK609629, MK609708, MK609762~MK609768 | <8>  8 |
| Chenjia Village, Heilongjiang, China | CJ | 127.56°E, 44.89°N | BOC039~041, BOC043~045, BOC049~051, BOC053, BOC056, BOC061, BOC062, BOC069~098, BOC106~113, BOC141~147, BOC149, BOC159~187 | MK609566~MK609568, MK609570~MK609579, MK609634, MK609635, MK609705~MK609707, MK609709~MK609718, MK609773, MK609774 | <15>  87 |
| Xiaoling, Heilongjiang, China | XL | 127.18°E, 45.21°N | BOC188, BOC189, BOC192~200, BOC203~205, BOC207, BOC209~219, BOC221~229, BOC231, BOC232, BOC235, BOC237~255 | MK609596~MK609605, MK609640~MK609643, MK609735~MK609744, MK609779~MK609782 | <14>  58 |
| Maoer Mountain, Heilongjiang, China | ME | 127.50°E, 45.27°N | BOC001~015, BOC017~035, BOC100~102, BOC475, BOC477~479, BOC481~484, BOC487~491, BOC494~500, BOC502~524, BOC526~529, BOC531, BOC534, BOC535, BOC537~539, BOC541, BOC542, BOC544~548, BOC550, BOC604~637 | MK609580~MK609587,  MK609636, MK609637,  MK609719~MK609726, MK609775, MK609776 | <10>  132 |
| Weihe, Heilongjiang, China | WH | 128.21°E, 44.49°N | BOC297~333 | MK609606~MK609609, MK609653~MK609657, MK609745~MK609748, MK609792~MK609796 | <10>  37 |
| Weihu Mountain, Heilongjiang, China | WHS | 129.29°E, 44.81°N | BOC066~068, BOC256~269 | MK609588~MK609595, MK609638, MK609639, MK609727~MK609734, MK609777, MK609778 | <10>  17 |
| Sandaoguan, Heilongjiang, China | SDG | 129.31°E, 44.46°N | BOC430~458, BOC463~470 | MK609617~MK609620, MK609645~MK609649, MK609685, MK609686, MK609688, MK609756~MK609759, MK609784~MK609788, MK609824, MK609825, MK609827 | <12>  37 |
| Mudanfeng, Heilongjiang, China | MD | 129.43°E, 44.29°N | BOC393~417, BOC421~426, BOC428, BOC429 | MK609663~MK609668, MK609670~MK609676, MK609683, MK609684, MK609802~Mk609807, MK609809~MK609815, MK609822, MK609823 | <15>  33 |
| Dingning, Heilongjiang, China | DN | 131.07°E, 44.10°N | BOC593~603 | MK609677~MK609682, MK609799~MK609704, MK609816~MK609821, MK609839~MK609843 | <11>  11 |
| Lushui River, Jilin, China | LS | 127.84°E, 42.59°N | BOC335~364, BOC367~369, BOC371~378, BOC381, BOC383~388, BOC390~392, BOC585~592 | MK609610~MK609616, MK609658~MK609662, MK609749~MK609755, MK609797~MK609801 | <12>  59 |
| Linjiang, Jilin, China | LJ | 126.91°E, 41.81°N | BOC117~122 | MK609630~MK609633, MK609652, MK609699, MK609769~MK609772, MK609791, MK609838 | <6>  6 |
| Yulin Town, Liaoning, China | YL | 125.50°E, 41.03°N | BOC552~560, BOC562~584 | MK609621, MK609622, MK609650, MK609651, MK609687, MK609689~ MK609693, MK609760, MK609761, MK609789, MK609790, MK609826, MK609828~ MK609832 | <11>  32 |
| Kuandian, Heilongjiang, China | KD | 124.78°E, 40.73°N | BOC116, BOC128~132 | MK609694~ MK609698, MK609833~ MK609837 | <5>  6 |

Supplementary Table S2

|  | SL | CJ | XL | ME | WH | WHS | SDG | MD | DN | LS | LJ | YL | KD |
| --- | --- | --- | --- | --- | --- | --- | --- | --- | --- | --- | --- | --- | --- |
| Primer9H | 0 | 0.0422 | 0.0074 | 0.0166 | 0 | 0 | 0.0577 | 0.2931 | 0.1885 | 0.0215 | 0.1111 | 0.1161 | 0 |
| Primer12F | 0 | 0.0053 | 0.0155 | 0.0144 | 0.0119 | 0 | 0 | 0.0352 | 0 | 0 | 0 | 0.0216 | 0 |
| Primer13 | No Inf. | 0 | 0 | 0.0209 | 0 | 0 | 0 | 0 | 0.0250 | 0.0099 | 0 | 0.0256 | 0.1111 |
| Primer17 | 0.1239 | 0.0611 | 0.0344 | 0.0350 | 0 | 0 | 0.0866 | 0 | 0 | 0 | 0 | 0.0196 | 0 |
| Primer19 | No Inf. | 0.2125 | 0.0944 | 0.1717 | 0.3794 | 0.3416 | 0.1609 | 0.3206 | 0.1199 | 0.1722 | 0.3659 | 0.3269 | 0 |
| Primer141 | No Inf. | 0.1158 | No Inf. | 0 | No Inf. | 0 | 0.1859 | 0.4159 | 0 | 0.1684 | 0.3333 | 0.2767 | 0.2512 |
| Primer10F | 0.1239 | 0.0465 | 0 | 0.0327 | 0 | 0 | 0 | 0.1005 | 0.0334 | 0.0247 | 0.1374 | 0.0361 | 0.1111 |
| Primer23 | 0.0791 | 0.0994 | 0.1888 | 0.3683 | 0.2760 | 0.2338 | 0.4261 | 0.2328 | 0.3212 | 0.3341 | 0.2651 | 0.3558 | 0 |
| Primer105 | 0 | 0.1174 | 0.1214 | 0.0372 | 0.1016 | 0.0660 | 0 | 0.0509 | 0 | 0.0695 | 0.2071 | 0 | 0 |
| Primer42 | No Inf. | No Inf. | 0 | 0.0575 | 0 | 0 | 0.0920 | 0 | 0 | 0 | 0 | 0.1499 | 0 |
| Primer53 | 0 | 0 | 0.0362 | 0 | 0 | 0.0289 | 0.1200 | 0.0603 | 0 | 0 | 0 | 0.1033 | 0 |
| PrimerB14 | 0 | 0.0202 | 0 | 0.0497 | 0 | 0.0389 | 0.0336 | 0.1031 | 0 | 0.0341 | 0.0579 | 0.0223 | 0 |

Supplementary Table S3

|  | SL | CJ | XL | ME | WH | WHS | SDG | MD | DN | LS | LJ | YL | KD |
| --- | --- | --- | --- | --- | --- | --- | --- | --- | --- | --- | --- | --- | --- |
| Primer9H | 0.9207 | 0.1199 | 0.7491 | 0.0369 | 0.0130 | 0.8123 | 0.0149 | 0 | 0.0015 | 0.9046 | 0.0087 | 0 | 0.1592 |
| Primer12F | 0.8920 | 0.6374 | 0.0419 | 0.0205 | 0.5274 | 0.3177 | 0.8563 | 0.4132 | 0.3329 | 0.3349 | 0.0729 | 0.0011 | 0.7643 |
| Primer13 | - | 1 | 1 | 0.1645 | 0.6426 | 0.9694 | 0.8556 | 0.1406 | 0.4788 | 0.3367 | - | 0.5226 | 0.0647 |
| Primer17 | 0.3917 | 0.0953 | 0.0534 | 0.1639 | 0.6096 | 0.6579 | 0.0066 | 0.4702 | 0.0213 | 0.1536 | 0.1570 | 0.0522 | 0.1998 |
| Primer19 | - | 0 | 0.0759 | 0 | 0 | 0.0002 | 0.0304 | 0 | 0.0623 | 0 | 0.0211 | 0 | 0.0914 |
| Primer141 | - | 0.0001 | - | 1 | - | - | 0.0001 | 0 | - | 0 | 0.0204 | 0 | 0.0884 |
| Primer10F | 0.3753 | 0.3036 | 0.8324 | 0.0206 | 0.7875 | 1 | 0.5448 | 0.0827 | 0.3243 | 0.1102 | 0.0826 | 0.0114 | 0.0114 |
| Primer23 | 0.5298 | 0.0092 | 0.0022 | 0 | 0.0001 | 0.1046 | 0 | 0.5903 | 0.2444 | 0.0021 | 0.3413 | 0 | 0.0912 |
| Primer105 | 0.2369 | 0 | 0 | 0.0004 | 0.0005 | 0.2232 | 0.0364 | 0.2523 | 1 | 0.0122 | 0.1018 | 1 | 1 |
| Primer42 | - | - | 1 | 0.0109 | 1 | 1 | 0.0936 | 0.5286 | 0.6327 | 0.2686 | - | 0.0005 | 0.0469 |
| Primer53 | 0.4859 | 0.7882 | 0.0020 | 0.9145 | 0.9947 | 0.1451 | 0.0058 | 0.0249 | 0.7579 | 0.7100 | 0.7197 | 0.1642 | 0.0341 |
| PrimerB14 | 0.3910 | 0.0053 | 0.5002 | 0 | 0.9013 | 0.2186 | 0.3509 | 0.0203 | 0.2917 | 0.5711 | 0.4072 | 0.3665 | 0.3080 |

Supplementary Table S4

| Sample sites | *H* | *S* | *Hd* | *π* | *K* |
| --- | --- | --- | --- | --- | --- |
| SL | 2 | 1 | 0.250 | 0.00014 | 0.250 |
| CJ | 4 | 5 | 0.543 | 0.00049 | 0.876 |
| XL | 4 | 4 | 0.495 | 0.00039 | 0.692 |
| ME | 3 | 2 | 0.378 | 0.00022 | 0.400 |
| **Western population** | **9** | **11** | **0.443** | **0.00035** | **0.625** |
| WH | 5 | 8 | 0.800 | 0.00122 | 2.178 |
| WHS | 3 | 3 | 0.378 | 0.00034 | 0.600 |
| SDG | 4 | 7 | 0.742 | 0.00187 | 3.348 |
| MD | 5 | 10 | 0.705 | 0.00173 | 3.086 |
| DN | 5 | 10 | 0.709 | 0.00142 | 2.545 |
| LS | 5 | 7 | 0.667 | 0.00081 | 1.439 |
| LJ | 3 | 6 | 0.600 | 0.00134 | 2.400 |
| YL | 7 | 10 | 0.909 | 0.00216 | 3.855 |
| KD | 4 | 10 | 0.900 | 0.00246 | 4.440 |
| **Eastern population** | **30** | **32** | **0.869** | **0.00226** | **4.038** |
| ALL | 38 | 42 | 0.774 | 0.00182 | 3.245 |

Supplementary Table S5

| Sample sites | *Na* | *Ne* | *Ho* | *He* |
| --- | --- | --- | --- | --- |
| SL | 2.889 | 2.060 | 0.429 | 0.405 |
| CJ | 4.444 | 2.591 | 0.430 | 0.474 |
| XL | 4.667 | 2.582 | 0.491 | 0.520 |
| ME | 5.778 | 2.498 | 0.474 | 0.499 |
| **Western population** | **6.778** | **2.593** | **0.463** | **0.510** |
| WH | 6.111 | 3.929 | 0.677 | 0.656 |
| WHS | 5.000 | 3.472 | 0.626 | 0.621 |
| SDG | 6.222 | 3.425 | 0.589 | 0.629 |
| MD | 6.778 | 4.203 | 0.606 | 0.691 |
| DN | 4.778 | 3.038 | 0.606 | 0.626 |
| LS | 6.778 | 3.962 | 0.608 | 0.618 |
| LJ | 4.000 | 3.087 | 0.481 | 0.569 |
| YL | 7.444 | 4.005 | 0.600 | 0.643 |
| KD | 3.889 | 3.266 | 0.711 | 0.641 |
| **Eastern population** | **10.444** | **4.827** | **0.617** | **0.710** |
| ALL | 5.291 | 3.240 | 0.564 | 0.584 |
